# Reducing breast cancer recurrences with CDK4/6 inhibitor treatment of HR-positive, HER2-negative early stage breast cancer from a societal perspective

**DOI:** 10.1186/s13561-026-00809-w

**Published:** 2026-06-26

**Authors:** Stephanie Reitzinger, Thomas Czypionka, Gabriel Rinnerthaler

**Affiliations:** 1 Institute for Advanced Studies, Health Systems and Health Policy, Josefstädter Str. 39, 1080 Vienna, Austria; 2 Division of Oncology, Department of Internal Medicine, Medical University Graz, Auenbruggerplatz 15, 8036 Graz, Austria; 3 Austrian Breast and Colorectal Cancer Study Group (ABCSG), Nussdorfer Platz 8, 1190 Vienna, Austria

**Keywords:** Early stage breast cancer, CDK4/6 inhibitor, Ribociclib, Recurrence, Direct costs, Indirect costs, Austria

## Abstract

**Background:**

Hormone receptor (HR)–positive and HER2 receptor–negative breast cancer accounts for two thirds of all breast cancers. In case of distant recurrences, a non-curative strategy is usually implied. Hence, treatment options for early stage breast cancer to prevent metastatic recurrences are desirable. The aim of our study was to estimate the societal effect of reducing breast cancer recurrences by treating Austrian patients with stage II and III HR + /HER2 − breast cancer with CDK 4/6 inhibitors as an adjuvant treatment.

**Methods:**

We developed a 7-state Markov model with a time horizon of 30 years from a societal perspective and compared the follow-up direct and indirect costs between patients who were treated *with* and *without* a CDK4/6 inhibitor in the early stage. We distinguished between anatomic stage II/III and nodal status negative/positive subcohorts of patients. We used transition probabilities derived from the clinical trial NATALEE. Based on local registry data, we derived the Austrian female target population and matched it with the target patients of NATALEE.

**Results:**

We identified 1,340 Austrian target patients diagnosed in 2022. In the anatomical stage II and stage III subcohorts, approx. 735 and 236 metastatic cancer years could be saved over 30 years, respectively. We estimated that, thereby, societal costs of about €52 million (discount rate = 3%) associated with cancer recurrences could be saved in the 30-year follow-up. Almost 75% of cost savings resulted from reductions in health care costs and about 25% from productivity gains (incl. unpaid work).

**Conclusions:**

Treating stage II and III HR + HER2 − breast cancer patients with CDK4/6 inhibitors in Austria reduces metastatic breast cancer recurrences, resulting in considerable medical benefits and economic effects.

**Supplementary Information:**

The online version contains supplementary material available at https://doi.org/10.1186/s13561-026-00809-w.

## Background

About 1 in 11 women will develop breast cancer by age 75 in Europe [1]. It is the most common type of cancer in women, whereby hormone receptor positive (HR +) human epidermal growth factor receptor negative (HER2 −) breast cancer accounts for approximately two thirds of newly diagnosed breast cancers. Although it is a relatively less aggressive subtype [2], the prognosis of survival depends on a variety of factors, such as the anatomic stage, which is defined by tumor size, the number of affected lymph nodes and the existence of distant metastases, in addition to biological factors like the proliferation marker Ki-67, tumor grading and higher risk signatures in multigenomic test [3]. In case of stage IV cancer, i.e. breast cancer with distant metastases, treatment intends to delay the progression, but – in contrast to early breast cancer (stage I-III) –implies a non-curative strategy. Therefore, new treatment options for early stage breast cancer to prevent metastatic recurrences are desirable, but the potential medical and economic impact in the population is unknown.

Recent findings of the NATALEE clinical trial showed significant reductions of breast cancer invasive and metastatic recurrences with the treatment of ribociclib, a cyclin-dependent kinase (CDK) 4/6 inhibitor, in combination with nonsteroidal aromatase inhibitors (NSAI), for patients with HR +/HER2 − stage II and III breast cancer [4, 5]. This treatment combination was compared to standard of care (NSAI monotherapy). With the addition of ribociclib to NSAI the risk of disease recurrence could be lowered by 28.5% (hazard ratio 0.715) as compared to NSAI alone, at a median follow up time of 44.2 months. The absolute invasive disease-free survival (iDFS) benefit was 4.9% at time of data cut-off. These effects were mostly driven by a reduction in metastatic breast cancer recurrences [5]. So far, CDK 4/6 inhibitors have been introduced in Austria for the use in patients with HR +/HER2 − metastatic and early breast cancer with higher risk of recurrence as of 2016 and 2022, respectively.

Hence, the aim of our study was to estimate the economic effect of reducing breast cancer recurrence by treating Austrian stage II and III HR +/HER2 − breast cancer patients with a CDK 4/6 inhibitor as an adjuvant treatment, based on the findings of the NATALEE trial [4, 5]. We developed a Markov model over a time horizon of 30 years comparing direct medical and indirect costs from work reduction of paid and unpaid work, disability, and death between the standard-of-care (i.e., before the approval of CDK4/6 inhibitors for early breast cancer stages, adjuvant nonsteroidal aromatase inhibitor alone)—and the treatment-group (adjuvant ribociclib plus adjuvant nonsteroidal aromatase inhibitor). Our results eventually showed the estimated saved metastatic cancer years and the associated saved costs of cancer recurrences from the societal perspective for Austria, based on a female patient cohort diagnosed in 2022.

## Methods

### Model overview and intervention

**Fig. 1:**
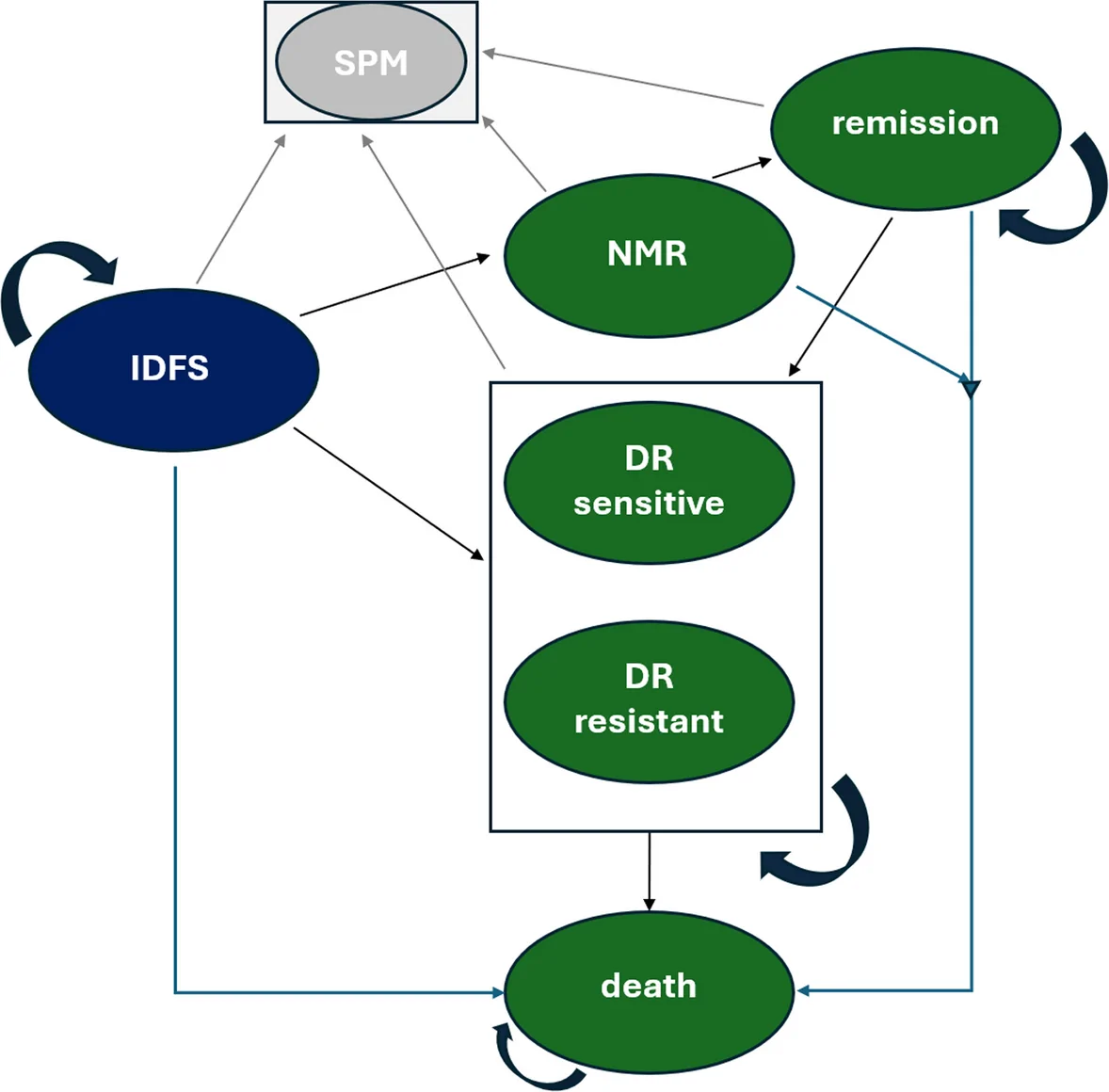
A simplified schematic depicting of the model structure: Markov model with 7 health states. IDFS: invasive disease-free survival; NMR: non-metastatic recurrence; DR sensitive: distant metastatic recurrence endocrine therapy sensitive; DR resistant: distant metastatic recurrence endocrine therapy resistant; SPM: secondary primary malignancy; cost-relevant health states are highlighted in green

We developed a Markov model to estimate the effects resulting from adjuvant CDK4/6 inhibitor treatment of stage II and III HR +/HER2 − breast cancer patients on breast cancer recurrences and the associated subsequent cost savings (i.e., the cost analysis starts with breast cancer recurrence). In the case of breast cancer recurrence, we distinguished between five health states. Our model is illustrated in Fig. 1; similar structures have been used in published former decision analyses [6, 7]. The cost-relevant health states included (i) non-metastatic recurrence (“NMR”), (ii) “remission”, (iii) distant metastatic recurrence endocrine therapy sensitive (“DR-sensitive”), i.e., those with recurrence > 12 months after the end of ET, or (iv) distant metastatic recurrence endocrine therapy resistant (“DR-resistant”), i.e., those with recurrence ≤ 12 months from the end of ET, and (v) “death”. Those with recurrence ≤ 12 months from the end of ET were assumed to enter the ET-resistant substate, while those with recurrence > 12 months after the end of ET were assumed to enter the ET-sensitive substate. The “NMR” health state is a temporary state (i.e., a tunnel state) where patients remain for 1 year and transition to the “remission” health state if they do not experience another event such as death or metastatic recurrence. In addition, a secondary primary malignancy (“SPM”) was modelled as an absorbing state (i.e., patients leave the model). In Fig. 1, the health states which were relevant in our cost analysis are highlighted in green color.

The model was implemented in Stata 18.0. Each cycle lasted 1 year (without half-cycle correction), and, in our results, we evaluated costs based on saved deaths and cancer-years over 10, 20, and 30 years, respectively, from societal perspective. Costs were discounted at a rate of 3% according to the recommendation of the European Commission for economic analyses for Austria [8].

**Fig. 2:**
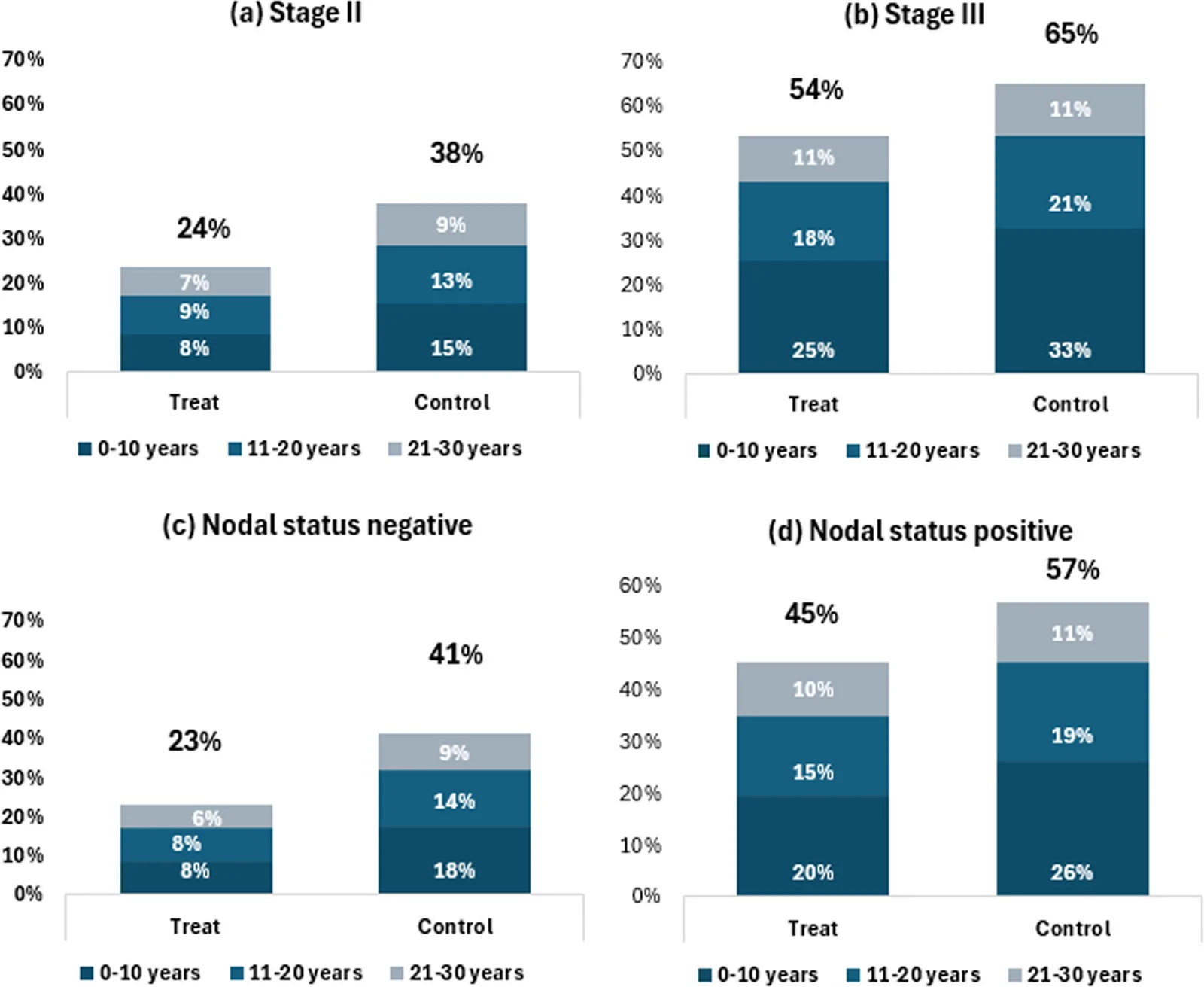
Incidence rate of metastatic cancer recurrence for anatomic stage II, stage III, nodal status negative, and nodal status positive treatment and control groups after 10, 20, and 30 years. The probabilities for cancer recurrence are derived from the NATALEE trial data with 44.2 months median duration of follow-up, and based on the exponential distribution

The intervention in our analysis was the adjuvant treatment for early stage HR +/HER2 − breast cancer consisting of ribociclib at a standard dose of 400 mg per day for 3 weeks, followed by 1 week off, for 3 years according to NATALEE trial design in addition to the standard-of-care endocrine therapy, which comprises nonsteroidal aromatase inhibitors (letrozole at a dose of 2.5 mg per day or anastrozole at a dose of 1 mg per day for ≥ 5 years) [5]. The NATALEE trial design is summarized in Table 2 in the supplementary material. The corresponding effects on metastatic cancer incidence are summarized in Fig. 2 for anatomic stage II and stage III cancer patients up to 30 years after the first diagnosis. Figure 2 further distinguishes by the underlying distributions that were employed to estimate the projected risk of recurrence based on the trial data with a median follow-up duration of 44.2 months [5].

Thereby, IDFS transition probabilities (transition to SPM, NMR, DR, and death) were estimated based on parametric survival distributions fitted to patient-level IDFS failure time data from the NATALEE trial. Given patient-level data from NATALEE, the IDFS hazard rate and the distribution of IDFS events by type (i.e., model health states) were multiplied to determine the probabilities of transitioning from IDFS to another health state in each model cycle. The probabilities of transitioning from IDFS to the Death state were adjusted for Austria’s general population mortality, with sex- and age-specific lifetables applied as a floor. Transition probabilities from the NMR state to death were assumed to be same as that from IDFS state. DR substates (ET-sensitive and ET resistant) were modelled as absorbing states based on data of previous ribociclib appraisals in the metastatic breast cancer setting.

According to the best-fitting distribution for the respective long-term projections (based on the Bayesian Information Criterion), we defined our main model on the probabilities for cancer recurrence resulting from the exponential distribution. Besides our main model, we further applied Weibull, Log-logistic and Gamma distributions (Figure A2) and reviewed the cost differences in the sensitivity analyses of our model. All these parametric models showed comparable visual agreement with the Kaplan–Meier curves. The fitted distributions remained within the 95% confidence intervals throughout follow-up, with no distribution demonstrating a superior visual fit compared with the others. We have not applied a half-cycle correction because transition probabilities were derived from continuous-time parametric survival hazards.

In the main model, the treatment with CDK4/6 inhibitor as adjuvant therapy decreases the probability for metastatic cancer recurrence from 15% to 8% in anatomic stage II and from 33% to 25% in anatomic stage III patients ten years after the initial early breast cancer diagnosis (Fig. 2). After 30 years, the probability decreases from 38% to 24% in anatomic stage II and from 65% to 54% in anatomic stage III patients, and from 41% to 23% in nodal status negative and from 57% to 45% in nodal status positive patients, respectively (Fig. 2).

### Patient population

We derived the Austrian target population newly diagnosed in 2022 consistent with the female target patients of the NATALEE trial using the national cancer registry 2022 [9] as the basis. The distributions regarding the anatomic stages, nodal status and age were drawn from the Clinical Tumor Register (Klinisches Tumor Register, KTR) of the Austrian Association for Gynecological Oncology (AGO), which contained a sample of 10,383 Austrian breast cancer patients diagnosed between 2015 and 2023 from hospitals of different Austrian regions [10], and from the breast cancer clinical register data of the university hospital Graz, which contained 3,881 patients diagnosed between 2006 and 2022 [11]. Table 1 shows the Austrian HR +/HER2 − female breast cancer cohort of 2022 who match with the NATALEE target population.

**Table 1:** HR +/HER2* − *breast cancer patients in Austria diagnosed in 2022 according to the NATALEE population [11]

	# of female patients	notes and sources
HR +/HER2 −	4,070	66.8%^a^ of total breast cancers^b^
without metastases	3,791	93%^c^
ECOG < 2	~ 3,602	~ 95%^d^
anatomic stage II	1,189	33%^a, c^
anatomic stage II (NATALEE)	980	82% of stage II ^c^
anatomic stage III	360	10%^c^
Cohort for the analysis	1,340	

^a^data from the KTR cohort, ^b^[10], ^c^data from the Graz cohort, ^d^[11]

Comparable with other countries [2], in Austria, about two thirds of total breast cancer cases account for the HR +/HER2 − subtype of which 93% are without metastases at the time of first diagnosis. Because patients with frailty and comorbidities were excluded from the trial we used the ECOG Performance Status as an exclusion criterion [12]. Accordingly, we excluded 5% of patients [13], who assumingly are patients with, for instance, clinically significant, uncontrolled heart disease, HIV, pregnancy, breast feeding, receiving or has received systemic corticosteroids ≤ 2 weeks, impairment of gastrointestinal function or gastrointestinal disease. Next, we assumed that one third are anatomic stage II patients and 10% are anatomic stage III patients. According to specific NATALEE definition of anatomic stage II target patients, we excluded further 18% of anatomic stage II patients. The specific exclusion criteria can also be found in Table 2 of our supplementary material. Finally, the cohort for our analysis consisted of 1,340 HR +/HER2 − female Austrian patients who were diagnosed in 2022 and who come into consideration to be treated with CDK4/6 inhibitors in the early stage of breast cancer.

**Table 2:** Characteristics of the Austrian HR +/HER2 − female breast cancer cohort diagnosed in 2022

total	1,340
< 40	54 (4%)
40–44	80 (6%)
45–49	161 (12%)
50–54	80 (6%)
55–59	147 (11%)
60–64	134 (10%)
65–69	147 (11%)
> = 70	536 (40%)
anatomic stage II	980 (73%)
anatomic stage III	360 (27%)
node neg	482 (36%)
node pos	858 (64%)

The age distribution assumed is based on the KTR anatomic stage II and stage III cohort 2022 and the division into anatomic stages and nodal status negative/positive is based on the Graz cohort

Hence, the cohort for our analysis consisted of 1,340 HR +/HER2 − patients (Table 2). Assumed 73% (980 patients) of our cohort were diagnosed with anatomic stage II and 27% (360 patients) with anatomic stage III cancer according to AJCC; assumed 36% (482 patients) of our cohort were diagnosed with node negative and 64% (858 patients) with node positive breast cancer. These shares differed between real world Austrian (Table 2) and NATALEE trial population [14], but we addressed these differences by modelling separate analyses for each of these four subcohorts. Moreover, the median age of early breast cancer patients was higher in the Austrian target population than in the clinical trial (Table 2), which could cause some bias towards the probability of death when using the clinical trial data for our analysis. However, we suppose that such bias is of minor importance for our analysis in which we focused mainly on recurrent breast cancer patients and not on the general population.

### Model assumptions for the treatment of cancer recurrences

The treatment schemes of our study were based on the guidelines of the AGO Breast Committee [3] and information from clinical practice, reflecting the state of breast cancer treatment in 2024. By following real-world data and recommendations from the Austrian Breast & Colorectal Cancer Study Group (ABCSG) [15], we defined two models—*Model A* and *Model B*. Thereby, our two models represent differences in actual treatment schemes as well as treatment differences between clinical breast cancer centers. The treatment of metastatic breast cancers may vary between cancer centers, because the treatment might depend on the use of innovative versus long established medications, regional differences in the composition of patients, or differences in the clinician’s specialization (gynecologists and oncologists). Furthermore, the treatment decision also depends on the clinical presentation (e.g. visceral crisis) and the endocrine-sensitivity status of the patients. In the case of metastatic breast cancer recurrences the recommendations have led us to define the following assumptions for *Model A* and *Model B*, respectively:

*Model A* for metastatic breast cancer recurrence:

1. 85% of patients receive a CDK4/6 inhibitor with *endocrine* treatment over two years followed by *endocrine and targeted* therapy for an additional 6 months as 1 st and 2nd line therapy, respectively. The remaining 15% of patients receive chemotherapy for 12 months
2. In the next step, 75% receive chemotherapy for 6 months
3. Followingly, 50% receive antibody–drug conjugates for 6 months. ^*^of the initial cohort who started 1L therapy (i.e. remaining patients who are alive and receive therapy [16])

*Model B* for metastatic breast cancer recurrence:

1. As first-line (1L) therapy, 85% of patients receive a CDK4/6 inhibitor with *endocrine* treatment over two years, 10% of patients receive chemotherapy for 12 months and 5% of patients receive no therapy (e.g. due to a patient’s reduced performance score, patient’s preference)
2. In second-line (2L), 80% receive further treatment: out of which 80% receive *endocrine and targeted* therapy and 20% receive chemotherapy for 6 months
3. In third-line (3L), 62%^*^ receive further treatment: out of which 50% receive *endocrine and targeted* therapy and 50% receive chemotherapy for 9 months. ^*^65% of the initial 95% who started 1L therapy (i.e. remaining patients who are alive and receive therapy [16])

Each treatment consisted of various options of medications. *First, endocrine* therapy in combination with a CDK4/6 inhibitor included, based on data of the Graz cohort, Anastrozole (42%), Letrozole (47%), Exemestane (11%) in the health state “DR sensitive”, and Fulvestrant (50%) and Tamoxifen (50%) in the health state “DR resistant”. Thereby, we assumed—based on data of NATALEE (Figure A1)—that no patients were in the “DR sensitive” state within six years after the first breast cancer diagnosis, because all patients received aromatase inhibitors (AI) as an adjuvant treatment. Similarly, we considered the costs of CDK4/6 inhibitors for the treatment group only from the fourth year in our model as these patients were treated with CDK4/6 inhibitors during the first three years anyway. Second, subsequent *endocrine and targeted* therapy included – based on the recommendations we gained from Austrian clinical breast cancer centers—Capivasertib (40%), Elacestrant (30%), Alpelisib (6%), CDK4/6 inhibitor with Fulvestrant (12%) and Everolimus with Exemestane (12%) in each model. We assumed that within the first 6 years about 15 percent of patients were pre-menopausal, who received Goserelin additional to endocrine therapy and Fulvestrant. Third, in model A, antibody–drug conjugates consisted of Sacituzumab-Govitecan (33%) and Trastuzumab-Deruxtecan (66%).

Furthermore, we integrated costs for inpatient hospital admissions, monitoring costs and the costs for psychosocial care for different health states. In the case of a non-metastatic cancer recurrence, based on the recommendations we gained from Austrian clinical breast cancer centers, we assumed that all patients had another surgery, 20% received a breast reconstruction and the other 80% patients with breast-conserving surgery received 15 units of radiation therapy, in each model. Moreover, we considered that patients with metastatic breast cancer receive 3 CTs per year and use additional inpatient healthcare services due to treatment-related reasons especially in advanced therapy lines, based on the clinicians’ advice. We assumed, therefore, that patients in the 2nd and subsequent therapy lines stay approximately 10 days per year in hospital (excl. palliative care) and in total 3 days during the first two years (i.e., during first line treatment). Based on the clinician’s observation and information, we also modelled that half of the patients with metastatic breast cancer recurrence receive psychosocial care once per month. In the case of death, we applied the costs of 7 days of palliative medical care in hospital and 7 days of hospice care for 70% of the patients at the end of life. For the other 30% of patients, we applied the highest care allowance (€1776 per month in 2022 [17]) for two weeks to partially capture formal or informal (palliative) care that is provided at home.

### Direct health care costs

To evaluate treatment costs, we used public medication prices of the pharmacist association price list and in- and outpatient DRG (diagnosis related group) points of the DRG-model 2025 with the base year 2022 [18]. For the latter, we weighted single treatments (e.g. different kinds of chemotherapy, radiation) to estimate the average cost of a breast cancer treatment unit (e.g. *chemotherapy, radiation*). The grouped and single treatments are described in more detail in Table 3 and its footnotes, respectively. The DRG system, however, does not distinguish between types of breast cancer. Therefore, we could not derive the sum of treatment units specifically for HR +/HER2 − patients or cancer stages, but only for breast cancer patients in general. We lastly multiplied the DRG points with 1.43 since DRG points only cover 70% of total costs of hospitals. The in- and outpatient prices, finally, reflect public as well as private health care expenditures (e.g., out-of-pocket self-contributions and private insurance payments). Secondly, the prices for medications which patients usually buy in pharmacies are expressed in public sick fund prices per month based on 30 days (Table 3). We calculated the average prices with generics and without parallel imports and the prices were drawn from the list of goods of the pharmacy association [19]. For pharmacy medications, it seemed more reasonable to use the most current prices, although the base year for our cost analysis was 2022.

**Table 3:** Average treatment unit costs (2022/2025), Austria

DRG data	€ per unit
breast surgery^a^	6533
breast reconstruction^b^	10,679
CT^c^	117
15 settings of radiation therapy^d^	3465
chemotherapy (per cycle)^e^	1116
psycho(social) therapy^f^	167
BC hospital day^g^	946
palliative and hospice care (14 days)^h^	9510
ADC (antibody–drug conjugates per cycle)^i^	8911

**pharmacist association price list** (March 2025)	**€ per month**
Anastrozole, Letrozole, Exemestane^j^	39
Tamoxifen	21
Fulvestrant	182
Alpelisib + Fulvestrant	3778
Capivasertib + Fulvestrant	7451
Elacestrant	9489
Everolimus + Exemestane	693
Goserelin	125
CDK 4/6 inhibitors^k^	2029

^a^partial mammary resection without axillary lymphadenectomy; with axillary lymphadenectomy; subcutaneous mastectomy without axillary lymphadenectomy, with axillary lymphadenectomy; total mastectomy without axillary lymphadenectomy, with axillary lymphadenectomy

^b^mammary reconstruction with implant; mammary reconstruction with free flap plasty

^c^price based on public health insurance fee schedule 2022[20]

^d^average costs of conventional linear radiation treatment and IMRT (40%), 3D image control as part of linear acceservice unitrator treatment

Paclitaxel < 150 mg/m^2^ (35%); Paclitaxel NAB (nanoparticservice unit albumin bound) (10%); Docetaxel < 100 mg/m^2^ (5%); Docetaxel > = 100 mg/m^2^ (4%); Eribulin (4%); Doxorubicin – liposomal (Caelix, Myocet) (3%); Carboplatin (1%); Gemcitabine (1%); Paclitaxel > = 150 mg/m^2^ (1%); Capecitabine (~ 35% [21]) based on the average public sickness fund price of the pharmacist association price list and on the dose of 4000 mg/day;

^f^initial psychiatric consultation and status assessment; clinical-psychological consultation; psychotherapeutic individual therapy

^g^average costs per inpatient hospital day with main diagnosis breast cancer

^h^resulting from 7 days in palliative care with average costs per day (€959 [22]]) and 7 days in hospice with average costs per day (€400[23]

^i^1/3 Sacituzumab-Govitecan, 2/3 Trastuzumab-Deruxtecan

^j^average public sick fund price of Letrozole (48%), Anastrozole (38%), Exemestane (13%)

^k^average public sick fund price of Abemaciclib, Palbociclib, and Ribocilib (based on an average dose of 477 mg [24] to reflect dose-linear prices)

### Indirect Costs

Breast cancer causes productivity losses either because the patients cannot attend their jobs while they are ill and seeking health care services, or they attend their jobs, but their capabilities are undermined because of the disease. Consequently, breast cancer patients are on sick leave more frequently and more likely to leave the work force due to disability [25]. In addition, the amount of non-paid care or voluntary work to society may also decline due to cancer-related sickness, disability, and early death.

In our model, we varied the assumptions regarding productivity losses from paid and unpaid work in three different *models I, II, III*.(Table 4) In *model I*, we only considered the productivity loss of women who participated in the labor market before their breast cancer recurrence. In two further models, we included the productivity loss from unpaid work of women below (model *II*) and above retirement age up to age 80 (model *III*), regardless of whether they had worked before their cancer recurrence or not.

**Table 4:** Models for the productivity loss

Model variations	
*I*.	“paid productivity loss up to 64 years”
*II*.	“paid and unpaid productivity loss up to 64”
*III*.	“paid and unpaid productivity loss up to 64 and unpaid productivity loss up to 80”

In all three models, we estimated the productivity loss from paid work, because of cancer-related labor force exits and cancer-related reduction of working hours, which are partially associated with cancer therapy and its side effects. Besides, former studies revealed a shift towards long-term sickness absences after the diagnosis of metastatic breast cancer [26, 27], and a possible “metastases”-effect on short-term sick leaves [25, 27]. Given the lack of data in this regard, however, we could only integrate sickness-related productivity losses by assuming that they are covered by related inactivity, disability, and hours reductions, which we estimated for each entire year (i.e., assuming patients exit or reduce working hours at the beginning of the year).

In our evaluation, the productivity loss is determined, first, by the average annual gross income (incl. employer contribution) of Austrian women in 2022 (€37,504) and an employment rate of 76.7% after the first cancer diagnosis. This estimate for the employment rate results from the employment rate of 81.1% in the Austrian female population in 2022 and the assumption that 5.4% [28, 29] of employed patients have not re-entered the labor force since their first cancer diagnosis. Then, after a *non-metastatic* and a *metastatic breast cancer recurrence,* we assumed the shares of patients who leave the work force are 6.6% [28] and 38% [30], respectively. To address the uncertainty of the data, we varied these shares using 0% [28] and 10.3% [28] for *non-metastatic breast cancer recurrences* and 17% [28] and 45% [31] for *metastatic breast cancer recurrences* in our sensitivity analyses. Moreover, for the remaining working patients, we assumed a 6.4% cancer penalty on income—based on Austrian data [32]—because of cancer-related working hours reduction. Since we had no specific data on metastatic cancer recurrence in this regard, we applied the average breast cancer penalty on the average income of the general female population as described above [33, 34]. In the case of death, we summed up the discounted productivity loss per death up to the age of 64 from paid work (plus up to 64 and 80 from unpaid work in model *II* and *III*, respectively).

In model *II* and *III*, we additionally estimated the productivity losses from unpaid work. We assumed that productivity unpaid work decreases half as much as paid work in case of cancer recurrence for all patients (employed, unemployed, inactive, and, in model *III*, retired women between 65 and 80 years). Thereby, we assumed that the women’s productivity decreases overall, but not as much in the unpaid work. In the case of death, we assumed the full productivity loss from paid work and unpaid work per year up to the age of 65 (and 80 in model *III* regarding unpaid work). We valued the loss of productivity from unpaid work with the product of the hourly wage of typical household services in private households in Austria (€15 [35]), the average hours per day to do care, household and voluntary work of adult women (3.8 h/day Table 4 in [36]), and 365 days (resulting in €20,805 per year). Since the unpaid labor supply will more likely be offset by elderly care needs with increasing age, we have set the age limit to 80.

If former employed patients did not return to work after cancer recurrence, we assumed that these patients receive disability pension until their regular retirement age of 64 [28]. In Austria, patients receive disability pension if they have paid at least 90 months of compulsory insurance contributions and their ability to work has declined to such an extent that it is less than half that of a healthy person [37]. In 2022, the yearly disability pension income of women was on average €15,302 [38].

## Results

**Fig. 3:**
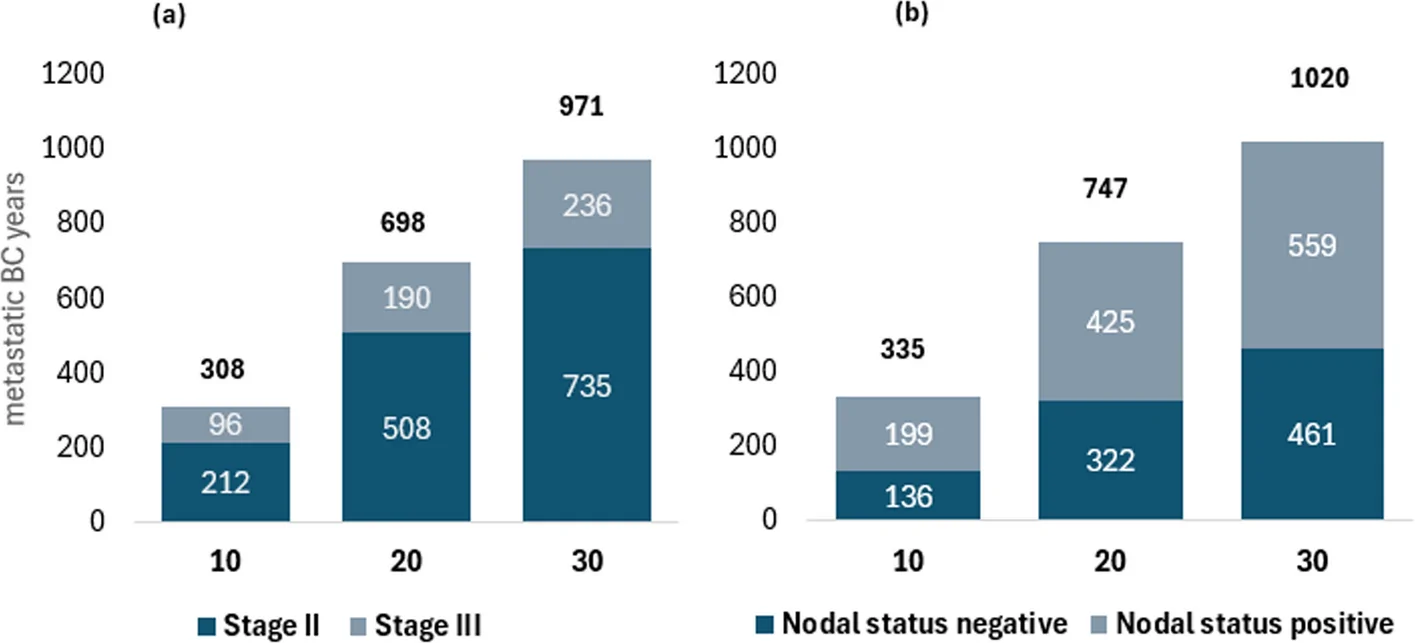
Saved metastatic breast cancer (BC) years over 10, 20, and 30 years, by different subcohorts The probabilities for cancer recurrence are derived from the NATALEE trial data with 44.2 months median duration of follow-up, and based on the exponential distribution

In our main analysis, we derived that the risk for metastatic breast cancer recurrences could be reduced by 37% in anatomic stage II, 17% in anatomic stage III, by 44% in nodal status negative, and by 19% in nodal status positive patients after 30 years (Fig. 2). Second, about 2% to 3.4% of the initial cohort of patients could be saved from a non-metastatic breast cancer recurrence. Third, metastatic cancer years could be reduced by 40% (735 years) in the anatomic stage II, by 20% (236 years) in the anatomic stage III, by 47% (461 years) in the nodal status negative, and by 23% (559) in the nodal status positive subcohort, respectively (Fig. 3). Thus, among the 1,340 Austrian patients (Table 2) who were diagnosed with HR +/HER2 − early stage breast cancer in 2022, it means that the incidence of metastatic cancer could be reduced by 15 percentage points, which are approximately 200 metastatic breast cancer recurrences.

Based on the treatment scheme of model A, our main results for anatomic stage II and stage III Austrian patients diagnosed in 2022 yielded €45.9 million, €51.3 million, and €52.2 million savings in follow-up costs in the models *(I), (II), and (III)* over 30 years, respectively*.* Correspondingly, for the cohorts of nodal status negative and nodal status positive patients, the estimated follow-up cost savings yielded €48.8 million, €54.2 million, and €55.1 million (Table 5)*.* Although the division of subcohorts lead to differences in the total sum of cost savings due to the underlying uncertainties of the transition probabilities, the cost savings across the different subcohorts show consistent values: the cost of cancer recurrence in the anatomic stage II and nodal status negative subcohorts was each reduced by about a third of the status quo long-term costs (i.e., the costs of the control group), and by nearly a fourth in the anatomic stage III and nodal status positive subcohorts, respectively (Table 5).

**Table 5:** Total cost reductions

model	years	Stage II	Stage III	Nodal status negative	Nodal status positive
		*n* = *980*	*n* = *360*	*n* = *482*	*n* = *858*
	10	11,171,412 (47%)	6,059,868 (34%)	6,752,824.5 (47%)	12,110,094 (34%)
*(I)*	20	25,527,060 (46%)	11,068,788 (28%)	15,673,888 (37%)	23,670,620 (26%)
	30	33,084,608 (31%)	12,787,724 (23%)	20,307,172 (40%)	28,446,128 (23%)
	10	12,776,831 (43%)	6,713,144 (31%)	7,506,537 (44%)	13,476,330 (32%)
*(II)*	20	29,236,344 (44%)	12,553,240 (27%)	17,646,776 (36%)	26,832,584 (25%)
	30	36,992,440 (31%)	14,346,340 (22%)	22,397,648 (34%)	31,773,520 (23%)
	10	13,004,650 (43%)	6,829,675 (31%)	7,656,436.5 (44%)	13,711,176 (32%)
*(III)*	20	29,726,948 (44%)	12,770,392 (27%)	17,967,550 (36%)	27,295,296 (25%)
	30	37,617,220 (31%)	14,598,928 (22%)	22,803,660 (34%)	32,329,960 (23%)

Total cost reductions based on model specifications A(I)- A(III) over 10, 20, and 30 years, by subcohorts (in €). The percentage refers to the control group’s total costs; the underlying transition probabilities are derived from the NATALEE trial data with 44.2 months median duration of follow-up, and based on the exponential distribution

In all subcohorts, up to 75% of total cost was saved by health care expenditure (incl. palliative and hospice care) and about 25% of saved costs came from paid and unpaid productivity gains (Table 6). Comparing, in addition, model (*I*) with model (*III)* in Table 5, we concluded that unpaid productivity gains made up about 13% of our total cost estimates (i.e. about the half of total estimated productivity gains).

**Table 6:** Health care cost reductions (in €)

	**Stage II** (*n* = *980)*		**Stage III** (*n* = *360)*	
	Model A *(III)* 30 years	Model B *(III)* 30 years	Model A *(III)* 30 years	Model B *(III)* 30 years
Total health care costs^a^	28.2	24.1	10.8	9.3
Health care costs *(% of total)*	*75%*	*72%*	*74%*	*71%*
Hospital admissions	*10%*	*12%*	*10%*	*11%*
CDK4/6 inhibitors	*17%*	*20%*	*22%*	*25%*
Endocrine/Targeted therapy	*30%*	*57%*	*29%*	*52%*
ADC	*32%*	*0%*	*30%*	*0%*
Chemotherapy	*6%*	*7%*	*6%*	*6%*
Other^b^	*5%*	*4%*	*3%*	*6%*

^a^in addition, palliative and hospice care expenditure account for about 1% of total costs

^b^e.g., psychosocial therapy, NMR-related costs (breast surgery, breast reconstruction, radiation therapy)

Health care cost reductions (in €) and cost shares for anatomic stage II and stage III subcohorts, by treatment schemes (model A and B). The underlying transition probabilities are derived from the NATALEE trial data with 44.2 months median duration of follow-up, and based on the exponential distribution

Furthermore, the total cost results were similar when comparing models A and B with different treatment schemes (Tables 5 and 6). Table 6 summarizes the underlying cost factors of health care expenditures in model A and model B for the anatomic stage II and stage III subcohorts. Thereby, the shares indicate the total costs’ sensitivity to each cost component. It has turned out that the results of model A depend most on prices of endocrine therapy and ADCs while the results of model B even more depend on the prices of endocrine/targeted therapy. All the other cost factors, including CDK4/6 inhibitors, hospitalizations and chemotherapy, had a similar impact on total health care costs in both models. Hence, as the disparities regarding prices between specific endocrine/targeted medications and antibody–drug conjugates, as well as regarding the proportion of patients receiving chemotherapy, were small, the cumulative cost reductions in model B were finally close to those in model A.

Finally, our findings provided a basis to roughly estimate the costs per patient or per metastatic cancer year. Costs per patient or year, thereby, depended on the model specification and on the underlying subcohort. The costs slightly differed between the subcohorts of our model not because of differences in the modelled treatment schemes but because of differences between their risk of breast cancer recurrence or death. Against this backdrop, we divided about €52 million by 200 metastatic cancer recurrences and gained approximately €195,000 (75%) health care expenditure (incl. palliative and hospice care) and €65,000 (25%) productivity loss per patient with a metastatic breast cancer recurrence, adding up to a societal impact of about €260,000 per breast cancer recurrence patient.

### Sensitivity analysis

To account for the uncertainty of our assumptions we conducted probabilistic and one-dimensional deterministic sensitivity analyses. In the probabilistic analysis, we explored the sensitivity of both model structure and parameters. We considered simultaneous fluctuations of various variables 1000 times: a random draw was taken from every distribution (exponential, Weibull, Gamma, log-logistic), prices varied within a range of 80% to 120% of the pharmacy purchase prices, the indirect cost factor parameters varied by base, low, and high values (i.e. the share labor force exits) and a random draw of a discount rate between 2 and 4 percent was taken. In the one-dimensional analysis, we varied single parameters: First, we applied the risk of breast cancer recurrence based on the Weibull and Gamma distribution. Second, we varied the assumptions on labor force exits (as described in the section “indirect costs”). Third, we applied the pharmacy purchase prices, which are usually about 5% lower than the public sick fund prices with some exceptions due to maximum margin regulations (25%−33% lower prices) [19]. Fourth, we varied the discount factor using 0% (the “raw” sum of costs), 2%, and 4%; the latter two should reflect differences in the time preferences of society. And fifth, we varied medication prices by 10% and 20% to consider potential price-models or rebates on the one hand, as well as potential future, more costly therapies on the other side.

**Fig. 4:**
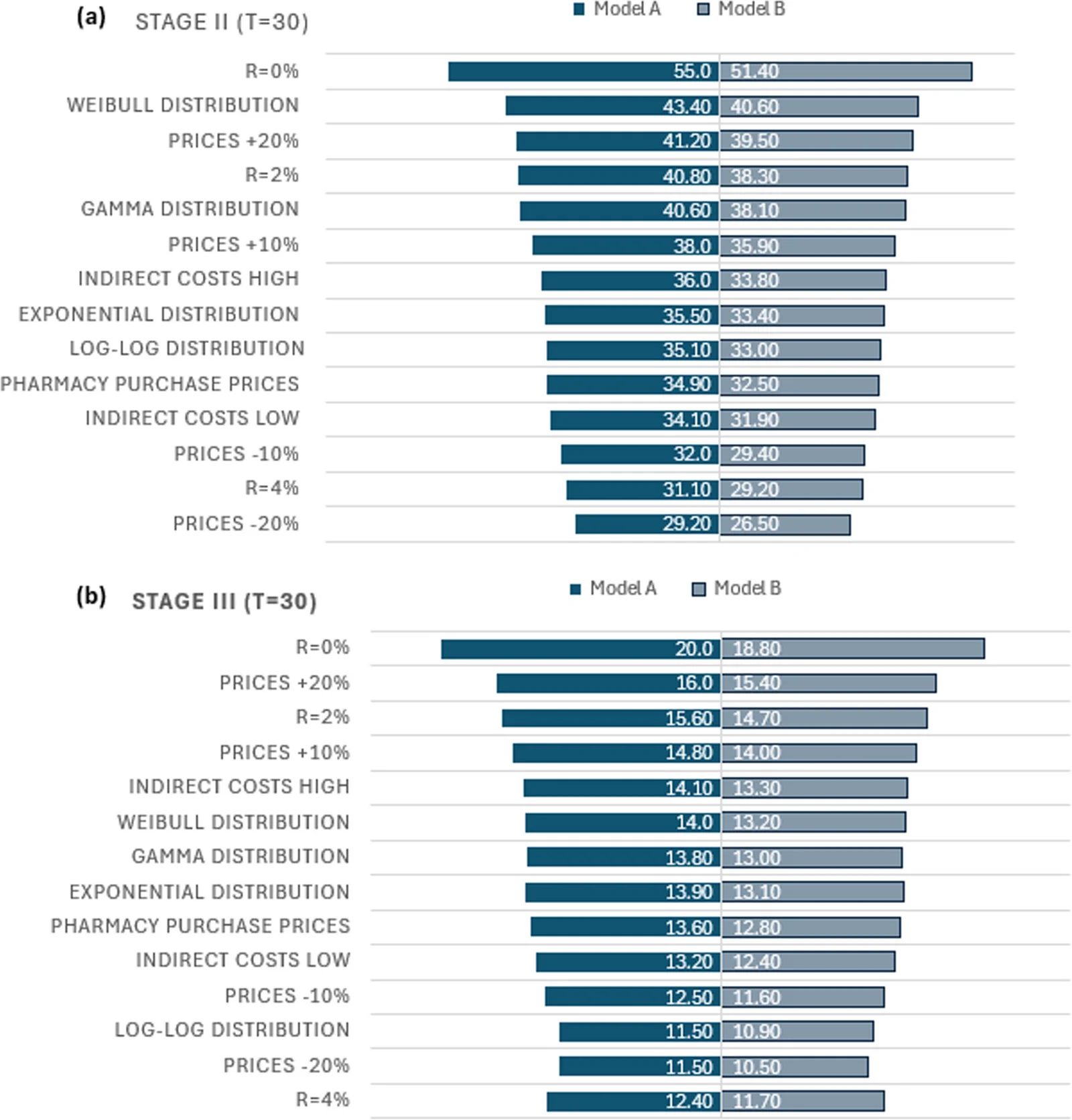
One-dimensional sensitivity analysis results for Model A and Model B (incl. unpaid work, over 30 years) for anatomic stage II and III cohorts (in € million). Variations regarding the risk of breast cancer recurrence are considered by variations in the distributions (“Exponential”, “Weibull”, “Gamma”); variations in direct costs are considered by price variations (“pharmacy purchase price”, “prices + 10%”, “prices −10%”, “prices + 20%”, “prices −20%”); variations in patients who return to work are considered in “indirect costs low” and “indirect costs high”

Accordingly, Fig. 4 summarizes the one-dimensional sensitivity of the estimated cost savings from reducing breast cancer recurrences for each subcohort, which result from model A and B including unpaid work up to the age of 80 and over a period of 30 years. Applying the risk of recurrence data based on the Weibull and Gamma distribution yielded mostly a rise in costs, while the log-logistic distribution yielded a decrease in costs. In comparison, the cost results appeared to be less sensitive to the assumptions on indirect costs and pharmacy prices. Applying a discount rate of 3% reduced the costs by about 30% to 40% compared to the raw sum of costs (r = 0%). Hence, we concluded that the forecast about the risk of breast cancer recurrence, medication prices, and discount rates were the most significant factors of uncertainty.

In addition, the model findings remained stable under the probabilistic sensitivity analysis. The probabilistic sensitivity analysis indicated that the mean outcome for stage II and stage III was €42.4 million (95% UI: 34.4–51.6) and €13.3 million (95% UI: 11.1–15.8), respectively, according to Model A, and, €36.9 (95% UI: 30.3–44.4) and €11.8 (95% UI: 9.9–13.8), respectively, according to Model B. The mean outcome for nodal negative and nodal positive was €25.6 million (95% UI: 20.6–31.3) and €37.8 million (95% UI: 30.6–45.7), respectively, according to Model A, and, €22.2 (95% UI: 18.1–26.8) and €32.5 (95% UI: 26.7–39.0), respectively, according to Model B.

## Discussion

Our study estimated the economic effect of treating Austrian HR +/HER2 − stage II and III breast cancer patients with a CDK 4/6 inhibitor as an adjuvant treatment, based on the findings of ribociclib of the clinical trial NATALEE [14]. We identified 1,340 female Austrian target patients in 2022. For this cohort, the results showed societal follow-up costs of at least €50 million (r = 3%) associated with breast cancer recurrences that could be saved over 30 years. Thereby, the costs of recurrence were reduced by about 30% in anatomic stage II and nodal status negative patients and reduced by about 20% in the anatomic stage III and nodal status positive subcohorts, respectively. These costs mainly resulted from the reduction of approximately 200 metastatic breast cancer recurrences and approximately 1,000 metastatic breast cancer years. Nearly 75% of the cost savings resulted from savings in health care expenditure and about 25% from productivity gains (incl. unpaid work).

We simplified our model by using data only from the NATALEE trial. In the monarchE trial, the efficacy of the CDK4/6 inhibitor abemaciclib in the adjuvant setting has turned out to be similar: The 4-year absolute improvement in iDFS was 4.9% and 6% compared with NSAI alone for ribociclib and abemaciclib, respectively. Still, differences in the outcomes could be explained by the differences in the trial population (i.e., NATALEE included a broader patient population), or specific pharmacological characteristics of the agents. Hence, in clinical practice, the choice between CDK4/6 inhibitors includes considerations on patient risk profiles, possible side effects, efficacy, treatment duration, and individual patient preferences [39].

Our findings may serve as a foundation for cross-country comparisons, bearing in mind that health expenditures are highly contingent on the country's context, its health care system, and the year of observation, owing to the rapid medical advancements in cancer treatment. Still, our estimate of health care expenditure, which yielded about €195,000 per HR +/HER2 − breast cancer recurrence, are quite comparable, for example, to a Canadian study which estimated approximately 257,000 in CAD 2023 per HR +/HER2 − patient in anatomic stage IV [40]. In Spain, for HR +/HER2 − metastatic breast cancer patients the average direct costs were estimated with €120,000 in 2016 [41]. Accordingly, with our study we contributed the case of Austria to this field of research studying the societal costs of breast cancer and the economic effects of new breast cancer treatments, especially with the focus on HR +/HER2 − breast cancer.

Moreover, the strength of our study is that we include a broad spectrum of data sources. We received aggregated data from the phase III clinical trial NATALEE. In order to approach generalizable findings for the real-world clinical landscape in Austria, we also collected clinician recommendations from three Austrian breast cancer centers and aggregated real-world data from clinical registries. In addition, we used information from the guidelines of the AGO Breast Committee [3] and findings of international health economic studies. Given the variety of the data we have gathered, we could address variations by data sources and the underlying uncertainties by using six different model specifications and 13 variations in the sensitivity analysis.

In the sensitivity analysis, it has turned out that our findings are sensitive to the underlying parametric distribution used to extrapolate IDFS, reflecting uncertainty in the shape of the recurrence hazard and associated costs beyond the observed trial period. While the exponential model assumes a constant hazard – which may be clinically implausible in early breast cancer, where recurrence risk typically changes over time and may decline or plateau – only minor differences were observed between distributions, such as the exponential and log-logistic models.

Moreover, our analysis relies on the assumption that relative treatment effects observed in NATALEE remain consistent between trial population and the real-world Austrian setting. We aimed to lower the differences by restricting the analysis to the real-world population who fulfilled the treatment criteria of the clinical trial and we conducted analyses specifically for sub-populations to adjust for nodal status and tumor stage. Nevertheless, differences, e.g. in the age distribution, exist.

We also recognize that assuming constant proportions for iDFS events, with the exception of transitions to the Death state which were adjusted for Austria’s general population mortality, may not fully reflect potential time-dependent changes in recurrence patterns. This structural assumption introduces uncertainty, particularly in extrapolation beyond the observed follow-up period.

Further limitations are that, first, we have left out several cost factors, which we had not enough data for, such as the costs related to care needs, costs of caregivers (e.g., impact on caregivers’ productivity), costs of treatment-related transportation [42], and generally the costs within the outpatient health care sector. Nevertheless, in Austria, cancer care is mainly provided in hospitals and outpatient clinics, as oncologists in private practice barely exist. Second, our results are based on average prices compiling different medications for each type of therapy. For instance, we had no information on the distribution of the types and doses of CDK4/6 inhibitors used, which differ in their prices per dose, and on the costs of medical examinations in this regard. Third, the costs of adverse events are approximated within our assumption on inpatient hospitalization, given that inpatient admissions of metastatic breast cancer patients are primarily treatment related [43]. While adverse events are usually treated by dose reductions (e.g., neutropenia) in the first-line treatment, we had no data to differentiate the inpatient admissions between therapy types in subsequent therapy lines.

Finally, post-recurrence treatment pathways might change after the introduction of adjuvant CDK 4/6 inhibitor use, treatment benefits may diminish over time, but not enough data has been available yet to observe such effects in the metastatic setting.

## Conclusion

We conclude that in Austria by treating early-stage HR + HER2 − breast cancer patients with CDK4/6 inhibitors a significant number of metastatic cancer recurrences could be avoided based on a lasting long-term impact over 30 years, reducing a substantial sum of subsequent societal costs each year. Because this study focused on the cost consequences following breast cancer recurrences and did not include the costs of initial adjuvant treatment, the analysis captures potential economic benefits from reduced recurrences but does not represent a full assessment of the entire care pathway or a cost-effectiveness evaluation. Future research needs to address how new medications for early-stage and advanced breast cancer affect productivity, employment and in- and outpatient health care visits of patients, and, data on the utility of new treatment options from the perspective of the patients. Moreover, it would be interesting to conduct a societal cost–benefit analysis for node-negative stage IIA patients with certain high-risk features in the near future.

## Supplementary Information

Supplementary Material 1.

## Data Availability

The datasets used and/or analysed during the current study are available from the corresponding author on reasonable request.

## Abbreviations

ADC: Antibody–drug conjugates
AI: Aromatase inhibitors
AGO: Austrian Association for Gynecological Oncology
BC: Breast cancer
CAD: Canadian dollar
CDK: Cyclin-dependent kinase
DR: Distant metastatic recurrence
DRG: Diagnosis related group
ET: Endocrine therapy
HR: Hormone receptor
HER2: Human epidermal growth factor receptor 2
iDFS: Invasive disease-free survival
KTR: Klinisches Tumor Register
(1L) (2L) (3L): First, second, third line therapy
NMR: Non-metastatic recurrence
NSAI: Nonsteroidal aromatase inhibitors
SPM: Secondary primary malignancy
