# Supplementary material for: Reducing breast cancer recurrences with CDK4/6 inhibitor treatment of HR-positive, HER2-negative early stage breast cancer from a societal perspective
Source: Health Econ Rev. 2026 Jun 26;16:103. doi: 10.1186/s13561-026-00809-w (PMC13595678; doi:10.1186/s13561-026-00809-w)
Supplement: Supplementary file 1 [file 13561_2026_809_MOESM1_ESM.docx]

**Additional Materials**

**Reducing Breast Cancer Recurrences with CDK4/6 Inhibitor Treatment of HR-Positive, HER2-Negative Early Stage Breast Cancer from a Societal Perspective**

Stephanie Reitzinger, PhD

[reitzinger@ihs.ac.at](mailto:reitzinger@ihs.ac.at)

Thomas Czypionka, MD, MSc

Gabriel Rinnerthaler, MD

**Table A1 Breast cancer incidence, prevalence, and mortality in Austria in 2022**

|  | incidence ^a^ | incidence per 100,000 ^a,b^ | prevalence ^a^ | prevalence per 100,000 ^a,b^ | mortality ^a^ | mortality per 100,000 ^a,b^ |
| --- | --- | --- | --- | --- | --- | --- |
| total | 6,161 | 84 | 89,930 | 1,220 | 1,605 | 21 |
| women | 6,096 | 162 | 89,188 | 2,362 | 1,588 | 42 |

Note: ^a^ [1]*,* ^b^ population>19 years [2]

**Table A2 NATALEE trial design** [3]

| **Treatment group** | **Control group** |
| --- | --- |
| **Inclusion criteria:** patients ≥18 years of age; histologically confirmed HR+/ HER2- early breast cancer according to local assessment stage II or III, at the time of surgery for patients who had not received adjuvant or neoadjuvant treatment, the “worst stage” was derived with the use of TNM staging at diagnosis and at the time of surgery for patients who had received adjuvant or neoadjuvant treatment. Patients with stage IIA disease with at least one lymph node involved. Patients who had no nodal involvement and a grade 2 tumor with a Ki-67 proliferation index of at least 20% or who were in a high genomic risk group, and patients with stage IIA disease with no nodal involvement and grade 3 tumors were eligible.  **Exclusion criteria:** Patients were ineligible if they had received a previous CDK4/6 inhibitor or if they had had clinically significant, uncontrolled heart disease, cardiac repolarization abnormalities, or both (patients could have received any adjuvant or neoadjuvant endocrine therapy for up to 12 months before randomization*: ~63%).*  Or patient with N0 and T2 (except G3; or G2 with Ki-67 ≥20% or high genomic risk) | |
| **Randomization** was stratified according to anatomical stage *(~40% stage II and ~60% stage III*), menopausal status *(~44% premenopausal women and men and ~56% postmenopausal women*) previous adjuvant or neoadjuvant chemotherapy *(~88% yes and ~12% no*), and geographic location *(~60% Western Europe*) | |
| **Nonsteroidal aromatase inhibitor** (NSAI): letrozole at a dose of 2.5 mg, administered orally once daily, or anastrozole at a dose of 1 mg, administered orally once daily, on a continuous schedule for 60 months (or longer at the discretion of the treating physician) | |
| **Ovarian suppression**: premenopausal women in both groups *(~25%)* received goserelin for gonadal suppression (at a dose of 3.6 mg, administered subcutaneously once every 28 days) | |
| **Ribociclib**: a dose of 400 mg, administered orally once daily for 21 consecutive days, followed by 7 days off, for a complete cycle of 28 days, administered for 36 months | - |

**Fig. A1**

Prevalence of metastatic breast cancer recurrences for anatomic stage II (above) and stage III (below) breast cancer in the treatment (left) and control group (right) (estimate from exponential distribution). Note: Transition probabilities have been derived from the clinical trial NATALEE (data cut off: April 2024)

**Fig. A2**

Incidence rate of metastatic breast cancer recurrence for anatomic stage II, stage III, Nodal status negative, and Nodal status positive treatment and control groups after 10, 20, and 30 years, by different model distributions

Note: Transition probabilities have been derived from the clinical trial NATALEE (data cut off: April 2024)

**Table A3 Employment rate of the female population in Austria 2022 ^a^, by age groups**

|  | 30-59 years | 30-49 years | 50-59 years |
| --- | --- | --- | --- |
| employed | 0.811 | 0.823 | 0.791 |
| inactive or unemployed | 0.181 | 0.162 | 0.214 |

Note: *^a^* [2]

**Table A4 Sensitivity analysis results for Model A and Model B (incl. unpaid work, over 30 years) for nodal status negative and nodal status positive cohorts (in € million)**

|  | **Nodal status negative** (*n=482)* | | **Nodal status positive** (*n=858)* | |
| --- | --- | --- | --- | --- |
|  | Model A *(III)*  30 years | Model B *(III)*  30 years | Model A *(III)*  30 years | Model B *(III)*  30 years |
| Exponential distribution | **22.8** | **20.3** | **32.3** | **29.0** |
| Gamma distribution | *26.1* | *23.1* | *32.7* | *29.3* |
| Weibull distribution | *27.8* | *24.6* | *39.3* | *34.3* |
| Log-log distribution | *22.1* | *19.6* | *26.9* | *24.3* |
| Indirect costs low | *21.9* | *19.3* | *30.9* | *27.6* |
| Indirect costs high | *23.1* | *20.6* | *32.9* | *29.5* |
| Pharmacy Medication Prices | *21.2* | *19.7* | *30.2* | *28.2* |
| Prices +10% | *23.1* | *21.8* | *32.8* | *31.1* |
| Prices +20% | 25.2 | 24.1 | 35.5 | 34.2 |
| Prices -10% | 19.4 | 17.8 | 27.7 | 25.6 |
| Prices -20% | 16.4 | 16.0 | 23.7 | 23.2 |
| r=0% | 35.6 | 31.4 | 48.1 | 42.7 |
| r=2% | 26.3 | 23.3 | 36.7 | 32.8 |
| r=4% | 19.9 | 17.7 | 28.7 | 25.8 |

**Literature**

1.	Statistik Austria: Cancer, https://www.statistik.at/en/statistics/population-and-society/health/cancer

2.	Statistik Austria: STATcube – Statistische Datenbank., http://www.statistik.at/web_de/services/statcube/index.html (2019), Accessed 15 August 2022

3.	Slamon, D., Lipatov, O., Nowecki, Z., McAndrew, N., Kukielka-Budny, B., Stroyakovskiy, D., Yardley, D.A., Huang, C.-S., Fasching, P.A., Crown, J., Bardia, A., Chia, S., Im, S.-A., Ruiz-Borrego, M., Loi, S., Xu, B., Hurvitz, S., Barrios, C., Untch, M., Moroose, R., Visco, F., Afenjar, K., Fresco, R., Severin, I., Ji, Y., Ghaznawi, F., Li, Z., Zarate, J.P., Chakravartty, A., Taran, T., Hortobagyi, G.: Ribociclib plus Endocrine Therapy in Early Breast Cancer. N. Engl. J. Med. 390, 1080–1091 (2024). https://doi.org/10.1056/NEJMoa2305488
